# Performance of expanded diagnostic criteria for APECED in independent cohorts and implications for earlier diagnosis

**DOI:** 10.1172/jci.insight.203021

**Published:** 2026-07-22

**Authors:** Elise M.N. Ferré, Joseph Pechacek, Monica M. Schmitt, Taura Webb, Heather Moorman, Thomas DiMaggio, Princess Barber, Vasielios Oikonomou, Stacey R. Rose, Peter D. Burbelo, Lindsey B. Rosen, Amy P. Hsu, Jennifer Stoddard, Shakuntala Rampertaap, Sergio D. Rosenzweig, Anjali Rai, Maria Teresa Magone, Chantal Cousineau-Krieger, Niki M. Moutsopoulos, Pamela J. Gardner, Heidi H. Kong, Leslie Castelo-Soccio, Ariane Soldatos, Katherine R. Calvo, Meryl Waldman, Behdad Afzali, Stefania Pittaluga, David Kleiner, Steven M. Holland, Kevin Fennelly, Jill Rothschild, Bryce A. Seifert, Magdalena Walkiewicz-Yvon, Theo Heller, Karen Winer, Michail S. Lionakis

**Affiliations:** 1 Fungal Pathogenesis Section, Laboratory of Clinical Immunology and Microbiology (LCIM), National Institute of Allergy and Infectious Diseases (NIAID),; 2 Adeno-Associated Virus Biology Section, National Institute of Dental and Craniofacial Research (NIDCR),; 3 Immunopathogenesis Section, LCIM, NIAID,; 4 Immunology Service, Department of Laboratory Medicine (DLM), Clinical Center,; 5 Translational Hepatology Section, Liver Diseases Branch, National Institute of Diabetes and Digestive and Kidney Diseases (NIDDK),; 6 Consult Services Section, National Eye Institute,; 7 Human Barrier Immunity Section, Laboratory of Host Immunity & Microbiome, NIAID,; 8 Dental Consult Services, NIDCR,; 9 Cutaneous Microbiome and Inflammation Section, National Institute of Arthritis and Musculoskeletal and Skin Diseases,; 10 Pediatric Neurology Consultation Service, Office of the Clinical Director, National Institute of Neurological Disorders and Stroke,; 11 Hematology Laboratory Service, DLM, Clinical Center,; 12 Clinical Nephrology Section, Kidney Diseases Branch, NIDDK,; 13 Laboratory of Pathology, National Cancer Institute,; 14 Laboratory of Chronic Airway Infection, Critical Care Medicine and Pulmonary Branch, National Heart, Lung, and Blood Institute,; 15 Pediatric Hospitalist Section, Department of Pediatrics,; 16 Division of Intramural Research, NIAID, and; 17 Pediatric Growth and Nutrition Branch, National Institute of Child Health and Human Development, NIH, Bethesda, Maryland, USA.

**Keywords:** Autoimmunity, Clinical Research, Immunology, Autoimmune diseases, Genetic diseases, T cells

## Abstract

Autoimmune polyendocrinopathy-candidiasis-ectodermal dystrophy (APECED/APS-1) is a monogenic autoimmune disorder of impaired central tolerance classically diagnosed by the presence of 2 out of 3 classic triad manifestations: chronic mucocutaneous candidiasis, hypoparathyroidism, and adrenal insufficiency. However, many patients develop non-triad manifestations years earlier, delaying recognition and care. In 2016, we proposed expanded diagnostic criteria incorporating 3 early clinical manifestations — APECED rash, autoimmune enteritis, and enamel hypoplasia — based on observations in 35 North American patients. Here, we provide further support for the clinical utility of these expanded diagnostic criteria in independent cohorts of 57 American and 12 European patients enrolled in a prospective natural history study at the NIH. Across all cohorts, the expanded diagnostic criteria decreased the time to diagnosis by half relative to the classic diagnostic criteria. Patients exhibited an enrichment of early non-endocrine autoimmune manifestations, underscoring disease heterogeneity and the potential for developing organ-specific autoimmunity before endocrine failure. These findings demonstrate the clinical utility of the expanded APECED diagnostic criteria and support the notion that their adoption might enable earlier disease recognition and timely immunomodulatory therapy to improve long-term outcomes.

## Abstract 2

Expanded clinical criteria identify APECED earlier, enabling faster diagnosis, improved monitoring, and opportunities to prevent severe complications and guide targeted treatment.

## Introduction

Autoimmune polyendocrinopathy-candidiasis-ectodermal dystrophy (APECED), also known as autoimmune polyendocrine syndrome type-1 (APS-1) (OMIM, 240300), is a monogenic disorder caused by deleterious variants in the autoimmune regulator (*AIRE*) gene, which impair central immune tolerance (1–4). Disruption of thymic tolerance allows autoreactive T cells to escape into the periphery and into target organs, where excess T cell–derived interferon γ (IFN-γ) activity has been shown to promote multiorgan autoimmune injury and chronic mucocutaneous candidiasis (CMC) (5, 6). The classic triad manifestations of APECED include CMC, hypoparathyroidism, and adrenal insufficiency. Clinical diagnosis has relied on developing any 2 of these classic triad manifestations, which raises suspicion for APECED and triggers *AIRE* sequencing and type I IFN autoantibody measurement (7–11).

Although developing a classic diagnostic dyad is characteristic for APECED, patients most often complete such a dyad relatively late in the course of the disease, which delays diagnosis (12–15). Therefore, expanding the classic diagnostic criteria with non-classic triad manifestations that occur earlier and more frequently among children with APECED might facilitate earlier diagnosis and improve outcomes. To that end, we previously demonstrated in a cohort of 35 American patients that addition of the adjunct triad of urticarial eruption (termed APECED rash, a recurrent, non-pruritic maculopapular eruption described in detail elsewhere) (15–17), autoimmune enteritis–driven intestinal dysfunction (referred to as autoimmune enteritis onwards), and enamel hypoplasia (18) to the classic diagnostic criteria would have reduced the time to diagnosis by half (~4 years) and would have allowed for establishing the diagnosis before presenting with life-threatening adrenal crises or hypocalcemic seizures in approximately 50% of patients (12). Importantly, while numerous non-classic manifestations occur in APECED (collectively referred to here as non-classic triad manifestations, i.e., all manifestations outside the classic triad), the term “adjunct triad” refers specifically to the subset of 3 early-onset, high-frequency manifestations — APECED rash, autoimmune enteritis, and enamel hypoplasia — identified as most informative for earlier diagnosis. Since no European patients were included in that initial analysis, it remained unclear whether these findings would also be applicable to European patients with APECED or whether the expanded criteria would reproducibly accelerate diagnosis in additional, independent American cohorts.

Following our report, 4 retrospective analyses of Brazilian (13), Turkish (14), Indian (19), and Italian (20) APECED cohorts found that implementing our proposed criteria could have promoted earlier diagnosis, suggesting that these expanded criteria may have broader applicability across cohorts. We have since consecutively enrolled 69 additional patients with APECED from North/South America (*n* = 57) and Europe (*n* = 12) to our NIH IRB–approved protocol and performed uniform, prospective clinical and research evaluations (12). We sought to confirm the utility of incorporating the adjunct triad into the classic diagnostic criteria to accelerate diagnosis in both American and European APECED cohorts and to update clinical, genetic, autoantibody, and immunologic findings in all 104 enrolled patients. We found that implementing our proposed expanded diagnostic criteria (i.e., developing any diagnostic dyad within the combined classic and adjunct triad manifestations) reduced the time to diagnosis by at least half in both American and European patients. We confirmed that among the non-classic triad manifestations, the most common early-onset manifestations are within our initially proposed adjunct triad — APECED rash, autoimmune enteritis, and enamel hypoplasia. Our findings also show that earlier diagnosis by the expanded diagnostic criteria could improve screening and prevent acute life-threatening endocrine failure and facilitate earlier recognition and treatment of non-endocrine autoimmune end-organ damage.

## Results

### Demographic and genetic features in the 69 newly enrolled patients with APECED.

Between 2015 and 2020, we consecutively evaluated 69 new patients from 59 nonconsanguineous families with a clinical and/or genetic diagnosis of APECED (see Methods) from North and South America (*n* = 57; US, 49; Canada, 4; El Salvador, 2; Uruguay, 1; Argentina, 1) and Europe (*n* = 12; Ireland, 8; England, 1; Scotland, 1; Isle of Man, 1; Germany, 1) (Supplemental Table 1). Among the 57 American patients, 24 were male, 33 were female, and 29 (51%) were children. Among the 12 European patients, 5 were male, 7 were female, and 9 (75%) were children (Supplemental Table 1; supplemental material available online with this article; https://doi.org/10.1172/jci.insight.203021DS1).

The most common *AIRE* variant in both cohorts was c.967_979del13, followed by c.769C>T (Supplemental Table 2). We found no correlation between the time to development of any autoimmune manifestations and carrying c.967_979del in homozygosity, carrying c.976_979del in compound heterozygosity with another deleterious *AIRE* variant, or carrying no c.967_979del allele (Supplemental Figure 1), suggesting that these common *AIRE* genotypes are similar in functional impact and that factors other than genotype may play a larger role in determining the manifestations that develop in an individual with these variants.

### Enrichment of non-endocrine manifestations in the 69 newly enrolled APECED patients.

We systematically evaluated each patient as previously described (12) and catalogued all their clinical manifestations. Both American and European patients developed an average of 2 endocrinopathies, with hypoparathyroidism (77% vs. 83%) and adrenal insufficiency (70% vs. 67%) being most frequent (Tables 1 and 2), consistent with previous reports (7, 8, 12). Ovarian failure (30% of American females, 29% of European females), hypothyroidism (28% of Americans), testicular failure (25% of American males, 20% of European males), growth hormone deficiency (12% of Americans, 8% of Europeans), and type 1 diabetes (5% of Americans, 8% of Europeans) were observed with less frequency (Tables 1 and 2). Consistent with prior reports, the presence of 21-hydroxylase and thyroid autoantibodies correlated with an earlier onset of adrenal insufficiency and hypothyroidism, respectively (Supplemental Figure 2).

Consistent with our prior findings (12), both cohorts exhibited a broad expansion of non-endocrine autoimmunity. Specifically, American patients developed a median of 5 non-endocrine manifestations (mean, 5.4; range, 1–10) (Supplemental Table 3), developing a median of 2 (mean 2.7; range, 1–7) non-endocrine manifestations before their first endocrinopathy, with only 3 patients (4%) developing an endocrinopathy as their first APECED manifestation. The 3 most prevalent early-onset non-triad manifestations were enamel hypoplasia, autoimmune enteritis, and APECED rash, confirming the adjunct triad proposed in 2016 (Table 1) (12). Collectively, we observed over 20 distinct clinical manifestations in American APECED patients, with a median of 7 manifestations per patient (mean, 7.6; range, 1–15).

European patients had fewer disease manifestations overall (median, 6; mean, 5.5; range, 3–13), which may reflect their younger age at the time of NIH evaluation (mean age, 14 years; 75% pediatric) relative to American patients (mean age, 20 years; 51% pediatric). European patients also demonstrated an enrichment of non-endocrine autoimmunity, developing a median of 4 non-endocrine manifestations (mean, 3.5; range, 2–10) (Supplemental Table 3). Moreover, European patients developed a median of 3 (mean, 2.7; range 1–5) non-endocrine manifestations before their first endocrinopathy; only 1 patient (8%) developed an endocrinopathy as their first manifestation. Among early-onset, non-classic triad disease manifestations, our proposed adjunct triad manifestations of enamel hypoplasia, autoimmune enteritis, and APECED rash were the most prevalent (Table 2).

There were notable differences in the frequency of non-endocrine manifestations between American and European patients, especially decreased prevalence among European patients of autoimmune hepatitis (21), autoimmune gastritis, autoimmune keratoconjunctivitis, Sjögren-like syndrome, and autoimmune pneumonitis (22, 23) compared with American patients (Tables 1 and 2). No European patient had yet developed hypothyroidism, asplenia, or tubulointerstitial nephritis by the time of NIH evaluation (Table 2). Further follow-up of these patients as well as recruitment of additional European patients, including from Nordic countries, will help further elucidate whether such differences in the prevalence of non-endocrine autoimmunity may exist between American and European patients.

Among other manifestations, CMC occurred in 50 (86%) American and in 10 (83%) European patients, among whom all individuals developed oral thrush, followed by vulvovaginal (47.5% of females), esophageal (39.7%), and nail candidiasis (27.9%). We again observed an incompletely penetrant association between autoantibodies against type 17 cytokines and the presence of CMC. The frequency of any of the type 17 autoantibodies tested (i.e., IL-17A, IL-17F, and IL-22) did not significantly differ among patients with or without CMC (Supplemental Table 4). Although present in only 32% of all patients, autoantibodies against IL-17A correlated with the time to developing CMC; by contrast, autoantibodies against IL-17F and IL-22 did not (Supplemental Figure 2 and Supplemental Table 4). As with our 2016 report, we again noted an enrichment of the hexad of non-endocrine manifestations of APECED rash, pneumonitis, autoimmune enteritis, hepatitis, gastritis, and Sjögren-like syndrome (Tables 1 and 2). Autoantibodies against bactericidal/permeability-increasing fold-containing B1 (BPIFB1), but not the potassium channel regulator KCNRG, correlated with the time to development of autoimmune pneumonitis in the newly enrolled patients (Supplemental Figure 2).

### The expanded diagnostic criteria result in earlier recognition of APECED.

**Figure 1:**
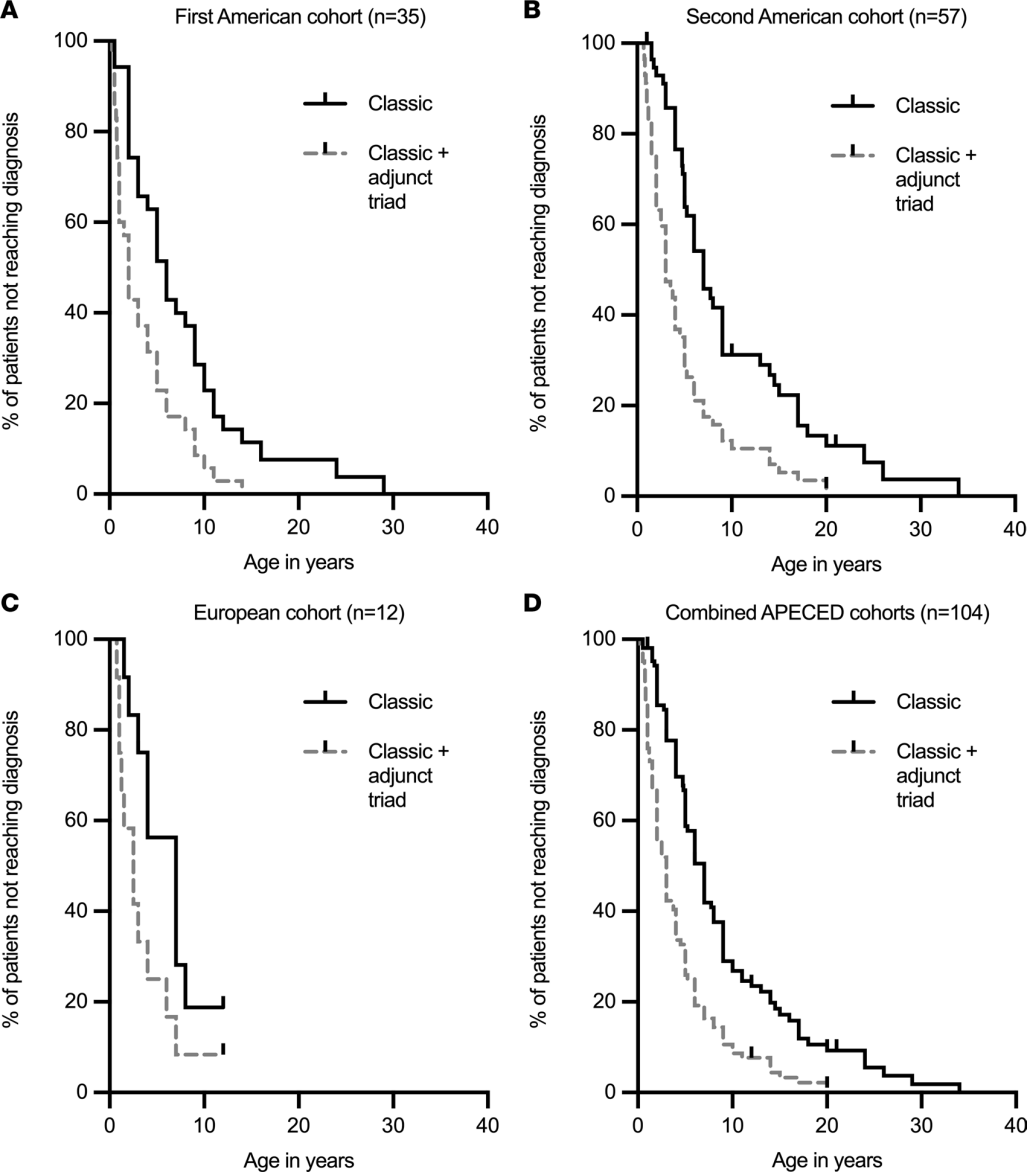
Incorporation of the adjunct triad manifestations of APECED rash, enamel hypoplasia, and autoimmune enteritis into the classic triad manifestations results in accelerated diagnosis across independent cohorts of American and European APECED patients. Shown in **A**–**D** are Kaplan-Meier curves comparing the time to APECED diagnosis between developing any diagnostic dyad among the classic triad criteria (CMC, adrenal insufficiency, hypoparathyroidism) or the expanded diagnostic criteria (classic triad plus adjunct triad manifestations of APECED rash, autoimmune enteritis, and enamel hypoplasia), as shown in the first 35 American patients (previously reported in and modified from Ferre et al., ref. 12) (**A**), a second independent cohort of 57 American patients (current study) (**B**), a cohort of 12 European patients (current study) (**C**), and in the entire cohort of all 104 APECED patients combined (**D**). Vertical hash marks on Kaplan-Meier curves represent individuals who have not yet met the designated clinical diagnostic criteria at the specified age during the time of evaluation at the NIH Clinical Center.

We analyzed the 57 American patients with the classic diagnostic criteria (i.e., developing any dyad among the classic triad manifestations of CMC, adrenal insufficiency, and hypoparathyroidism) and observed 48 (84%) reached a classic diagnostic dyad by a mean age of 8.8 (range, 1.5–34) years (Tables 1 and 3). Notably, the other 9 genetically confirmed patients with a mean age of 8.5 (range, 1–21) years had not yet developed a classic diagnostic dyad by the time of NIH evaluation. Importantly, 51 (89.5%) American patients developed an average of 2 (range, 1–5) non-classic triad manifestations before reaching a classic diagnostic dyad; among these, the most prevalent were the adjunct triad manifestations of APECED rash (24 patients, 42.1%), autoimmune enteritis (20 patients, 35.1%), and enamel hypoplasia (18 patients, 31.6%) (Supplemental Table 5); this distribution is consistent with our previous cohort analysis (12). Strikingly, implementing the expanded diagnostic criteria (i.e., developing any dyad among the classic triad manifestations of CMC, adrenal insufficiency, and hypoparathyroidism and the adjunct triad manifestations of APECED rash, autoimmune enteritis, and enamel hypoplasia) identified 56 (77.2%) American patients who could have been diagnosed approximately 4.5 years earlier (mean, 4.6 years; range, 0.67-20 years) relative to the diagnosis reached by classic diagnostic criteria, thus reducing the time to diagnosis by half, similar to our previously reported cohort (Figure 1, A and B, and Table 3). In 29 patients (50.8%), earlier diagnosis by the expanded diagnostic criteria would have provided a clinical benefit. Specifically, in 19 of them who presented with hypocalcemic seizures and/or adrenal crises before the time of diagnosis by the classic diagnostic criteria, these events could have been prevented with earlier diagnosis by the expanded diagnostic criteria. Moreover, 10 additional patients and 5 more among the aforementioned 19 patients would have been diagnosed earlier with the expanded diagnostic criteria, which may have allowed for earlier recognition and initiation of immunomodulation for non-endocrine autoimmune manifestations (i.e., autoimmune hepatitis, autoimmune pneumonitis, autoimmune keratoconjunctivitis) that developed before reaching a classic diagnostic dyad.

When evaluating the European cohort by the classic diagnostic criteria, we found 9 (75%) patients reached a classic diagnostic dyad by a mean age of 4.8 (range, 1.5–8) years, earlier than the American cohort (Tables 2 and 3). The other 3 genetically confirmed patients with a mean age of 9 (range, 3–12) years had not yet developed a classic diagnostic dyad at the time of NIH evaluation. Similar to the American patients, 10 (83.3%) European patients developed an average of 2 (range, 1–7) non-classic triad manifestations before reaching a classic diagnostic dyad, among which the 3 most common were our proposed adjunct triad manifestations of APECED rash (7 patients, 58%), enamel hypoplasia (5 patients, 42%), and autoimmune enteritis (3 patients, 25%) (Supplemental Table 5). Implementing our expanded diagnostic criteria would have reduced the time to diagnosis by half (mean, 2.8 years; range, 0.75–7 years) relative to the diagnosis reached by the classic diagnostic criteria (Figure 1C and Table 3). In 5 patients (41.7%) who manifested hypocalcemic seizures and/or adrenal crises before reaching a classic diagnostic dyad, these events could have been prevented with earlier diagnosis by the expanded diagnostic criteria. Among these patients, 2 could also have benefited from earlier recognition and treatment of autoimmune pneumonitis and autoimmune hepatitis, which occurred before reaching a classic diagnostic dyad.

When we combined the 69 patients from this study with our 35 previously reported American patients, implementing the expanded diagnostic criteria in the entire cohort (*n* = 104) also decreased the time to diagnosis by half relative to the time needed with the classic criteria (Figure 1D and Table 3); this beneficial effect may be underestimated because 13 patients (12.5%) with a current mean age of 9.1 years have not yet reached a classic diagnostic dyad but 11 of them have reached an adjunct diagnostic dyad at a mean age of 2.6 years. Two genetically confirmed patients, one 20-year-old Salvadoran male with CMC, autoimmune keratoconjunctivitis, growth hormone deficiency, and B12 deficiency and one 12-year-old Irish girl with CMC, alopecia areata, and autoimmune pneumonitis have not yet met a diagnostic dyad by either the classic or expanded diagnostic criteria.

We next aimed to validate our previous observation (12) that APECED rash, enamel hypoplasia, and autoimmune enteritis are indeed the 3 most appropriate manifestations, being prevalent early on, for inclusion in the expanded diagnostic criteria. For that, we analyzed the patients’ first 2 consecutive manifestations. Among the 57 American patients, the most common diseases occurring among their first 2 manifestations were CMC (70.2%) and APECED rash (33.3%), followed by autoimmune enteritis (21.1%), enamel hypoplasia and hypoparathyroidism (19.3% each), and autoimmune pneumonitis (12.3%) (Supplemental Table 6). As previously described, adrenal insufficiency occurred late (7, 12). Hence, the 3 most common non-classic triad diseases occurring among the first 2 manifestations were APECED rash, autoimmune enteritis, and enamel hypoplasia. Strikingly, in 39 patients (68.4%), their first 2 manifestations were among the expanded diagnostic criteria, whereas in only 5 patients (8.8%) were their first 2 manifestations among the classic diagnostic triad (*P* < 0.0001) (Table 4). European patients developed the same 6 manifestations as the most common among the first 2 that developed and the adjunct triad manifestations were the 3 most prevalent non-classic triad manifestations observed early on (Supplemental Table 6). As with American patients, in 10 European patients (83.3%) their first 2 manifestations were among the expanded diagnostic criteria manifestations, whereas in only 2 patients (16.7%) were their first 2 manifestations among the classic diagnostic triad (*P* = 0.003) (Table 4).

When considering the entire cohort of 104 patients, the first 2 consecutive manifestations were approximately 5 times more likely to be within the expanded diagnostic criteria (74 patients, 71.2%) than being within the classic diagnostic triad (14 patients, 13.5%; *P* < 0.0001) (Table 4). CMC and APECED rash were the most common first 2 manifestations, as previously reported (12), with CMC being most prevalent within the entire cohort. Importantly, CMC combined with any manifestation is most commonly seen (68 patients, 65.4%) within the first 2 consecutive manifestations in the entire cohort (Table 4), including in the aforementioned 12-year-old Irish girl with biallelic *AIRE* variants who developed CMC, alopecia areata, and autoimmune pneumonitis and has not yet met the classic or expanded diagnostic criteria (censored patient, Figure 1C). Notably, 28.8% of all patients developed combinations of the first 2 consecutive manifestations that were not both captured within either the classic or expanded diagnostic criteria. Therefore, a high index of suspicion for APECED is required in patients who develop serial autoimmune manifestations and particularly when these include any of the expanded diagnostic criteria manifestations of CMC, adrenal insufficiency, hypoparathyroidism, APECED rash, enamel hypoplasia, and autoimmune enteritis.

### Uncommon APECED manifestations in the entire cohort.

Evaluation of all 104 patients in our prospective NIH natural history study revealed several uncommon and/or previously under-recognized manifestations of APECED. Neurologic manifestations included episodic migraines in 12.5% of patients, a frequency similar to the average rate in population prevalence studies (24, 25), and seizure disorders in 5% diagnosed by EEG in the absence of hypocalcemia, hypoglycemia, or other metabolic abnormalities; most were well controlled, although one patient developed progressive cognitive decline, movement disorder, and intractable hiccups. Rare central nervous system autoimmune or structural findings included acute noninfectious encephalitis (*n* = 2), and one patient each with progressive encephalomyelitis with rigidity and myoclonus (PERM) (26), Arnold-Chiari malformation type 1, and hippocampal inversion, further expanding the previously reported central nervous system manifestations in APECED (27–30). Uncommon ocular diseases included recurrent blepharitis and chalazia (*n* = 5), amblyopia with esotropia in one individual with concurrent trisomy 21, and single cases of macular coloboma and retinopathy with nystagmus, optic nerve atrophy, and retinal dystrophy. Cardiac abnormalities were infrequent, including patent foramen ovale (*n* = 2) and one each of atrial septal defect (31) and tetralogy of Fallot. Pituitary adenomas were incidentally identified on MRI in 6 individuals (6%). Hepatobiliary disease included cholelithiasis in 14 patients (13.5%), consistent with prior reports, with a variable age of onset (range, 5–52 years) (32); 12 patients underwent cholecystectomy. Additional rare gastrointestinal manifestations included rectal prolapse due to severe autoimmune enteritis-associated diarrhea or constipation (*n* = 2), abdominal cocoon (33) in the context of autoimmune enteritis (*n* = 1), and postpartum sigmoid rupture (*n* = 1). Musculoskeletal manifestations comprised polyarthropathy with associated fever and myalgias in one 2-year-old child (*n* = 1) that responded to corticosteroids, and metaphyseal dysplasia in 2 individuals, one resolving spontaneously and another associated with genu varum of both lower extremities. Hematologic abnormalities were frequent, with iron deficiency anemia in 15 patients (14.5%) (34) and unprovoked hypercoagulability in 3 presenting as pulmonary emboli (*n* = 2) or recurrent deep vein thromboses (*n* = 1).

Malignancy was infrequent. We observed 2 gastric carcinomas (one each signet-ring and gastroesophageal junction adenocarcinoma) requiring total gastrectomy. Interestingly, both patients had intestinal metaplasia, prompting ongoing endoscopic surveillance in additional patients with premalignant gastric histological changes (35, 36). Oral or esophageal squamous carcinomas, described in other APECED cohorts (37–40), were not observed in our cohort, although 2 patients have had oral leukoplakia and remain under clinical surveillance.

Collectively, these findings further expand the recognized clinical spectrum of APECED, revealing diverse autoimmune, inflammatory, and structural complications that extend beyond the classic and adjunct triad manifestations. This underscores the importance of continued longitudinal evaluation to define disease penetrance, natural history, and mechanisms of organ-specific autoimmunity in APECED.

### Causes of mortality.

Among the 104 APECED patients, 10 (9.6%) died at a mean age of 32 years (range, 14–61 years). The principal causes of death were respiratory failure and/or infections associated with APECED-associated pneumonitis (*n* = 5), other infection-related complications (*n* = 2), endocrine failure (*n* = 2), and acute hepatic failure (*n* = 1).

Five deaths were attributed to advanced APECED pneumonitis complicated by irreversible bronchiectasis and secondary multidrug-resistant (MDR) bacterial infections. These included a 14-year-old boy with severe bronchiectasis who succumbed to acute respiratory failure and pneumonia; a 56-year-old man with a 40-year history of APECED pneumonitis who developed MDR *Pseudomonas aeruginosa* sepsis (41); a 61-year-old woman with longstanding pneumonitis and bronchiectasis who died of postoperative sepsis due to carbapenem-resistant *Pseudomonas aeruginosa* and *Klebsiella pneumoniae;* a 22-year-old woman who suffered from multiple recurrent catheter-associated bloodstream infections and bronchiectasis-associated bacterial pneumonias; and a 20-year-old woman who required extracorporeal membrane oxygenation for acute respiratory failure. In each case, chronic untreated autoimmune pneumonitis led to recurrent pulmonary infections and antibiotic exposure, promoting antimicrobial resistance and progressive respiratory decline.

Two patients died from complications of endocrine failure. A 28-year-old woman with known hypoparathyroidism was found deceased at home, possibly due to cardiac arrhythmia from severe hypocalcemia. Another patient, a man in his early 20s with adrenal insufficiency and type 1 diabetes died from combined adrenal crisis and hypoglycemia. A 42-year-old woman died in inpatient hospice care after developing encephalitis in the context of polyomavirus reactivation following immunosuppression with alemtuzumab and corticosteroids. A 24-year-old woman developed fulminant hepatic failure following a brief prodrome of elevated transaminases, suspected to be triggered by a viral infection on a background of unrecognized APECED-associated hepatitis. Finally, a 30-year-old woman hospitalized for septic shock developed fatal disseminated mucormycosis (42).

**Figure 2:**
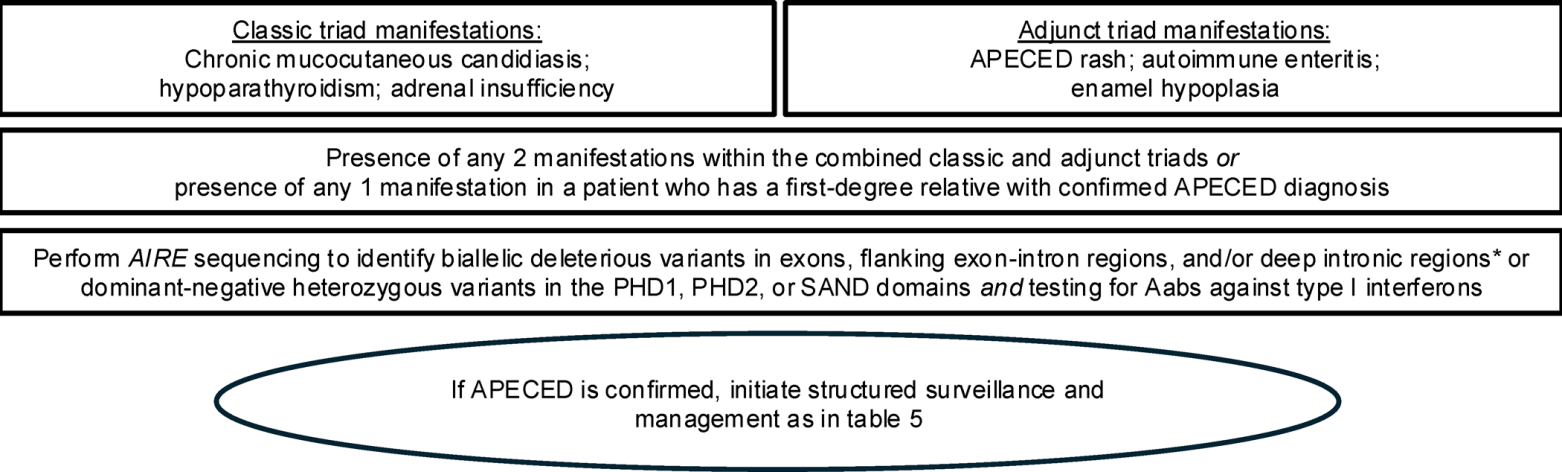
Proposed screening of prospective APECED patients. A proposed schema for screening practices to identify patients with APECED. AIRE, autoimmune regulator; PHD, plant homeodomain. AAbs, autoantibodies. *Note that pathogenic deep intronic variants may be missed by conventional genetic panels and whole exome sequencing. Whole genome sequencing, when available, may facilitate identification of such variants and may also reveal alternative genetic etiologies when *AIRE* variants are not detected.

Taken together, these findings underscore that preventable complications — most commonly pulmonary sequelae of autoimmune pneumonitis, followed by endocrine crises, hepatic failure, and severe systemic infections (21, 41, 43, 44) — remain the leading causes of mortality in APECED. These outcomes highlight the critical importance of early diagnosis, structured endocrine management, comprehensive surveillance and screening (Figure 2 and Table 5), and timely immunomodulatory therapy to prevent irreversible organ damage while not promoting immune compromise. Together, these approaches will improve long-term survival in APECED.

### Immunophenotyping of patient lymphocytes.

We had previously demonstrated an expansion of CD4^+^ naive T cells, autoreactive CD21^lo^CD38^lo^ B cells, and plasmablasts, and a contraction of NK cells in the peripheral blood of APECED patients (12). We performed immunophenotyping of peripheral blood lymphocytes in the entire cohort of 104 APECED patients and again found similar perturbations of these 4 lymphocyte populations (Supplemental Tables 7 and 8). In addition, we observed an expansion of CD8^+^ naive T cells and NKT cells in peripheral blood (Supplemental Tables 7 and 8). Together, these data define a characteristic immunophenotypic signature in APECED, marked by expansion of naive T cell subsets, dysregulated B cell compartments, and altered innate lymphocyte populations, reflecting the broad immune activation and loss of tolerance that underlie the disease.

### Serum CXCL9 levels are elevated across patient cohorts compared with healthy donors.

Given our previous observation that APECED is characterized by IFN-γ–driven inflammation with elevated serum and tissue CXCL9 (5, 6), we assessed serum CXCL9 levels across the initial American cohort, the newly enrolled cohort, and healthy donors. Both the initial and newly enrolled cohorts exhibited higher serum CXCL9 levels than healthy donors, consistent with a shared IFN-γ–driven inflammatory signature across cohorts (Supplemental Figure 3).

## Discussion

Our findings support the clinical utility of the expanded APECED diagnostic criteria incorporating APECED rash, autoimmune enteritis, and enamel hypoplasia across independent American and European cohorts. Collectively, these results confirm that inclusion of early non-endocrine manifestations substantially reduces time to diagnosis — by approximately half — relative to the classic diagnostic criteria, across genetically diverse populations.

APECED is not solely an endocrine disease but a systemic autoimmune disorder in which mucocutaneous, gastrointestinal, and ectodermal involvement frequently precedes endocrinopathy. The diagnostic shift we propose reframes APECED as a disorder of early, tissue-specific autoimmunity that evolves over time to multiorgan failure. Earlier diagnosis by these expanded diagnostic criteria enhances the opportunity to prevent life-threatening endocrine crises and to identify subclinical autoimmune organ damage while still reversible.

Mechanistically, these findings emphasize that AIRE deficiency produces a predictable but temporally heterogeneous sequence of autoimmune manifestations, likely shaped by environmental triggers and modifier genes. The reproducibility of the expanded diagnostic criteria across continents and genotypes underscores their robustness for clinical application. However, these findings reflect performance of the expanded criteria within confirmed APECED and do not constitute formal validation; further studies in broader and more heterogeneous populations, including individuals without biallelic *AIRE* variants, will be required to define diagnostic performance characteristics such as sensitivity and specificity. In addition, how individuals who meet expanded clinical criteria but lack identifiable *AIRE* variants evolve over time — whether representing early APECED, phenocopies, or a heterogeneous group of related immune-mediated conditions — remains to be determined. Broader sequencing approaches, including whole exome or genome sequencing, may therefore be informative in such patients by identifying alternative monogenic disorders that phenocopy aspects of APECED. Differences observed between cohorts should also be interpreted in the context of potential referral bias, including enrichment for more complex or severe phenotypes at a tertiary referral center.

Importantly, early identification opens the door for timely immunomodulation. Emerging data demonstrate that lymphocyte-targeted, IFN-γ–directed therapy can remit several APECED manifestations. Prophylactic immunomodulation in murine APECED models prevents autoimmune tissue destruction (5, 6, 45, 46). Together, these observations support a disease interception paradigm in which earlier diagnosis will enable targeted intervention before irreversible tissue injury.

Finally, our longitudinal cohort reveals a broader non-endocrine clinical spectrum and identifies preventable causes of morbidity and mortality, chiefly from untreated pneumonitis and hepatitis, endocrine crises, and infections. These insights argue for incorporation of the expanded diagnostic criteria into clinical practice guidelines and for continued longitudinal follow-up to define biomarkers of early disease activity and therapeutic responsiveness.

## Methods

### Sex as a biological variable.

Our study examined both female and male participants. We found a slight predominance of females to males in our cohort, which is consistent with other large, published cohorts of patients with APECED (20, 27).

### Study participants.

One hundred four consecutive APECED patients from North or South America and Europe were enrolled (2013–2020) on a NIAID IRB-approved protocol (ClinicalTrials.gov NCT01386437) and provided written informed consent; 35 of them were previously reported (2013–2015) (12) and 69 of them were evaluated between 2015 and 2020 and are described in the present study. Enrollment inclusion criteria were either (a) clinical diagnosis of APECED based on development of any 2 manifestations within the classic triad of CMC, hypoparathyroidism, and adrenal insufficiency or (b) confirmed genetic diagnosis of APECED via identification of biallelic *AIRE* mutations. All patients underwent uniform prospective evaluation as previously described (12). Healthy donors were enrolled in protocols approved by the NIH IRB committee and provided written informed consent for study participation. The study was conducted in accordance with the Helsinki Declaration.

### Genetic sequencing.

Whole exome sequencing and whole genome sequencing were performed on an Illumina sequencing system (minimum coverage, >95% at 20×), as previously described (6, 47, 48). Variant interpretation was performed according to ACMG guidelines (49) using the NIAID Bioinformatics and Computational Biosciences Branch–developed Genomic Research Integration System (GRIS). A custom comparative genome hybridization (CGH) array included 2408 genes involved in immunity (Gene Ontology terms containing immun*) and noncoding exons from 15 RNA genes or pseudogenes. Overall, greater than 99% of the targeted exons are covered by at least 3–4 probes and each of the 2408 genes had 1 probe approximately every 10 kb, while the entire genome backbone had 1 probe approximately every 55 kb, thus enabling copy number variation analysis at the individual exon or gene level and genome-wide.

### Autoantibody detection.

A multiplex particle-based approach was used to detect immunoreactivity against cytokines (GM-CSF, IL-12, IL-17A, IL-17F, IL-22, and IL-23) and IFNs (IFN-α, IFN-β, IFN-γ, and IFN-ω) as previously described (12) and were analyzed on a Bio-Plex X200 instrument (Bio-Rad). Luciferase immunoprecipitation systems (LIPS) measured autoantibodies against the lung antigens BPIFB1 and potassium channel regulator KCNRG (22) as described previously (12, 50). Autoantibodies against thyroid peroxidase and thyroglobulin were measured by chemiluminescence, while those against 21-hydroxylase were measured by immunoabsorption assay (12); these tests were performed in the NIH Clinical Center Department of Laboratory Medicine.

### Lymphocyte immunophenotyping.

Lymphocyte immunophenotyping was performed in peripheral blood mononuclear cells (PBMCs) of 100 participants with APECED (age range 2–66 years). Whole blood was collected in EDTA tubes and red cells were lysed with BD FACS Lysing Solution (BD Biosciences). Staining was performed and acquisition of flow data was as previously described (12). A same-day complete blood count with white count differential was used to quantify cell numbers from the FACS plot percentages. Samples from 40 adult healthy donors (age range 23–62 years) were collected following the same procedure and were used as a control.

### Serum CXCL9 measurement.

Patient sera were available for analysis in 31 patients from the initial cohort, 65 patients from the newly enrolled cohort, and 47 healthy donors. CXCL9 was measured by ELISA using a CXCL9 DuoSet kit (R&D Systems, DY392), as per the manufacturer’s instructions.

### Statistics.

Log rank tests were employed to compare time to development of a clinical manifestation by autoantibody presence and specific *AIRE* mutant alleles. Log rank tests were used since patients were of different ages thus had different lengths of time in which the clinical manifestation could present. Although we acknowledge that many comparisons were made, we consider a *P* value of less than 0.05 to be an indication that a variable would potentially be considered for further investigation. Comparisons of the frequency and/or absolute numbers of different lymphocyte subsets between APECED patients and healthy donors were performed using an unpaired, 2-tailed *t* test or Mann-Whitney *U* test using GraphPad Prism 10.0 and are presented as mean and range. A *P* value of less than 0.05 was considered statistically significant. As these are purely research generating results, no adjustment for multiple comparisons was made. CXCL9 serum levels in the initial and newly enrolled APECED cohorts and in healthy donors were compared using a Kruskal-Wallis test with Dunn’s multiple comparisons.

### Study approval.

This research was conducted under the IRB-approved protocols NCT01386437 and NCT03206099. Written consent was obtained from patients prior to undergoing any research procedures.

### Data availability.

All data in the article are included in the Supporting Data Values file.

## Author contributions

EMNF, JP, MMS, and MSL wrote the manuscript. EMNF, JP, MMS, TW, SRR, AR, MTM, CCK, NMM, PJG, HHK, LCS, AS, KRC, MW, BA, SP, DK, KF, JR, BAS, MWY, TH, SRR, KW, SMH, and MSL evaluated patients and provided clinical patient care. EMNF, JP, MMS, TW, HM, TDM, PDB, SRR, and MSL coordinated the conduct of clinical research protocols. VO, PDB, LBR, JS, SR, and SDR conducted experiments. EMNF, JP, MMS, TW, SRR, and MSL acquired data. EMNF, JP, MMS, TW, SRR, APH, SP, and MSL analyzed data. MSL designed the study.

## Conflict of interest

The authors have declared that no conflict of interest exists.

## Funding support

This work is the result of NIH funding, in whole or in part, and is subject to the NIH Public Access Policy. Through acceptance of this federal funding, the NIH has been given a right to make the work publicly available in PubMed Central.

- Intramural Research Program of the NIH.

## Supplementary Material

Supplemental data

Supporting data values

## Figures and Tables

**Table 1:**
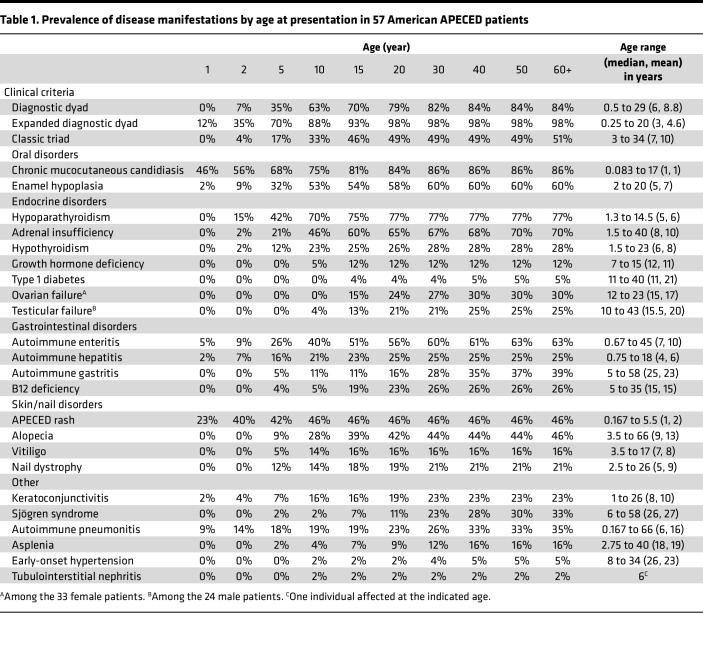
Prevalence of disease manifestations by age at presentation in 57 American APECED patients

**Table 2:**
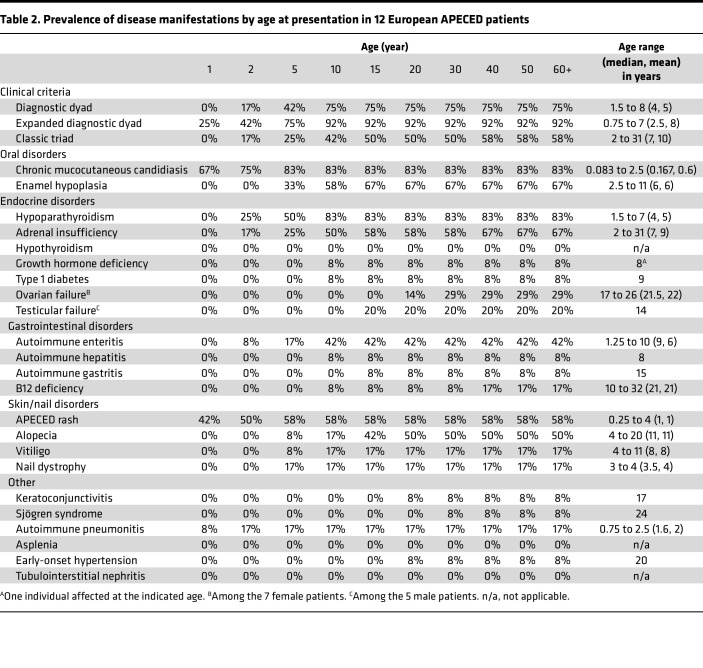
Prevalence of disease manifestations by age at presentation in 12 European APECED patients

**Table 3:**
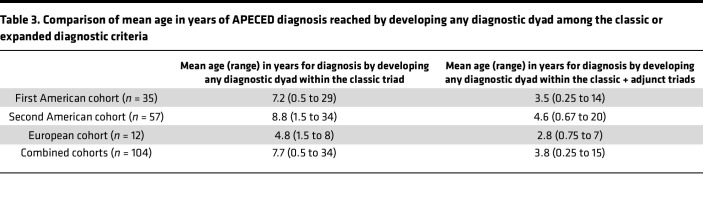
Comparison of mean age in years of APECED diagnosis reached by developing any diagnostic dyad among the classic or expanded diagnostic criteria

**Table 4:**
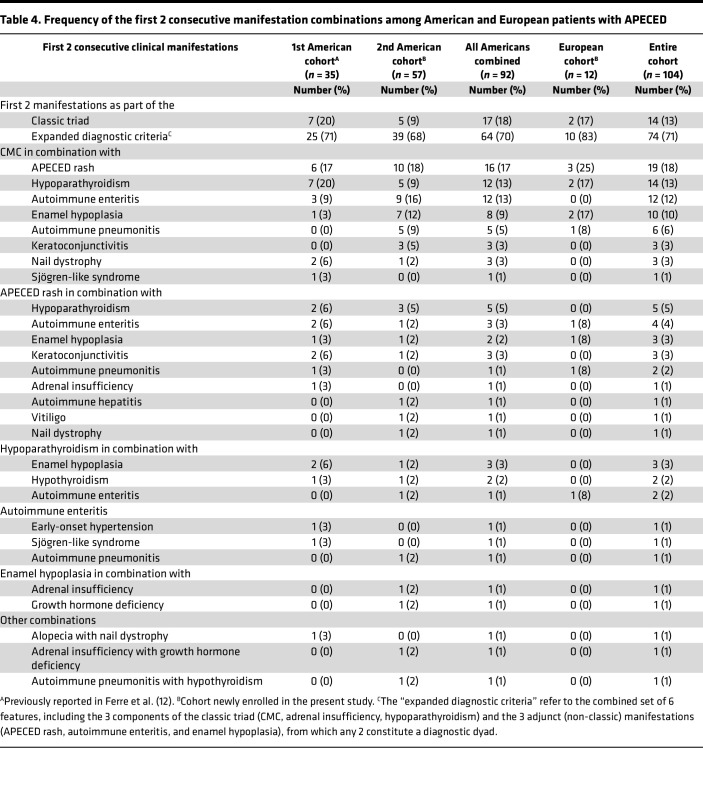
Frequency of the first 2 consecutive manifestation combinations among American and European patients with APECED

**Table 5:**
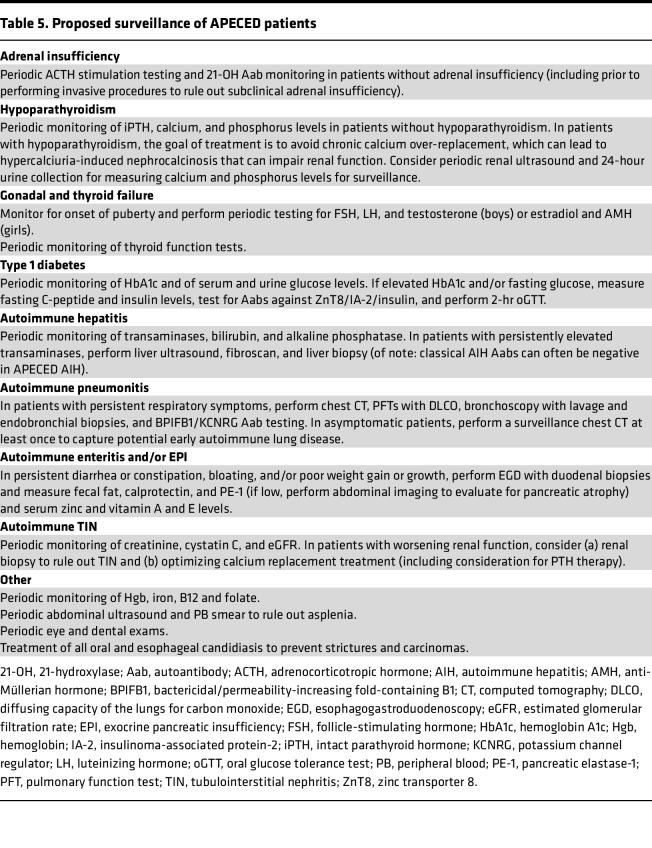
Proposed surveillance of APECED patients

## Version 1 07/22/2026

Electronic publication
