# Supplementary material for: Performance of expanded diagnostic criteria for APECED in independent cohorts and implications for earlier diagnosis
Source: JCI Insight. 2026 Jul 22;11(14):e203021. doi: 10.1172/jci.insight.203021 (PMC13461156; doi:10.1172/jci.insight.203021)
Supplement: Supplementary file 1 [file jciinsight-11-203021-s304.pdf]

List of Supplemental Material

1. Supplemental Table 1: Demographic characteristics of APECED patients included in this study
2. Supplemental Table 2: Frequency of mutant *AIRE* alleles amongst the American and European APECED patients included in the present study.
3. Supplemental Table 3: Frequency of endocrine and non-endocrine manifestations in APECED patients from the Americas, Europe, and combined geographic regions.
4. Supplemental Table 4: Summary of the presence of autoantibodies (AAB) to IL-17A, IL-17F, and IL-22 in the newly enrolled American and European cohort, their frequency in patients with or without chronic mucocutaneous candidiasis (CMC), and their association with the presence of CMC. n = 68 as AAB data was not available on one patient.
5. Supplemental Table 5: Frequency of non-classic triad clinical manifestations developing before APECED patients reach a classic diagnostic dyad.
6. Supplemental Table 6: Distribution of diseases amongst the first two consecutive manifestations in American and European APECED patients.
7. Supplemental Table 7: Absolute number of lymphocyte subsets in the peripheral blood of APECED patients and healthy donors.
8. Supplemental Table 8: Percent of lymphocyte subsets within corresponding lymphocytes in the peripheral blood of APECED patients and healthy donors.
9. Supplemental Figure 1. Time to development of APECED manifestations by the presence of the c.967_979del variant in the new cohort (n=69).
10. Supplemental Figure 2. Time to development of APECED manifestations by the presence of autoantibodies.
11. Supplemental Figure 3. Serum CXCL9 levels are elevated across patient cohorts and compared with healthy control.

Supplemental Table 1: Demographic characteristics of APECED patients included in this study

	First American cohort* (n=35)	Second American cohort** (n=57)	All Americans combined (n=92)	European cohort** (n=12)	Total cohort (n=104)
Number (%) of females	21 (60)	33 (58)	54 (59)	7 (58)	61 (59)
Number (%) of males	14 (40)	24 (42)	38 (41)	5 (42)	43 (41)
Mean age (range) in cohort	23 (6 to 63)	20 (2 to 66)	21 (2 to 66)	14 (3 to 32)	20 (2 to 66)
Number (%) of pediatric patients (<18 years)	16 (46)	29 (51)	45 (49)	9 (75)	54 (52)
Mean (range) age in pediatric patients	11 (6 to 17)	9 (2 to 17)	10 (2 to 17)	10 (3 to 15)	10 (2 to 17)
Number (%) of White / non-Hispanic***	31	44	75	12	87
Number (%) of White / Hispanic***	4	9	13	0	13
Number (%) of Asians***	0	1	1	0	1
Number (%) of patients reporting multiple races / non-Hispanic***	0	1	1	0	1
Number (%) of patients reporting multiple races / Hispanic***	0	2	2	0	2
**Residence outside of the US**					
Argentina	0	1	1	0	1
Canada (Ontario)	1	2	3	0	3
Canada (Manitoba)	0	1	1	0	1
Canada (Quebec)	0	1	1	0	1
Colombia	1	0	1	0	1
El Salvador	0	2	2	0	2
England	0	0	0	1	1
Germany	0	0	0	1	1
Isle of Man	0	0	0	1	1
Ireland	0	0	0	8	8
Scotland	0	0	0	1	1
Uruguay	0	1	1	0	1
**Residence in the US**					
Alabama	0	1	1	0	1
Arkansas	0	1	1	0	1
Arizona	0	1	1	0	1

California	3	1	4	0	4
Connecticut	1	0	1	0	1
Delaware	0	2	2	0	2
Florida	2	3	5	0	5
Georgia	2	1	3	0	3
Illinois	1	1	2	0	2
Louisiana	1	0	1	0	1
Kansas	0	1	1	0	1
Massachusetts	0	4	4	0	4
Maryland	1	1	2	0	2
Michigan	1	0	1	0	1
Minnesota	1	0	1	0	1
Mississippi	1	0	1	0	1
Missouri	1	3	4	0	4
Montana	0	1	1	0	1
New Hampshire	1	0	1	0	1
New Jersey	2	1	3	0	3
New York	3	12	14	0	14
Ohio	2	2	4	0	4
Oregon	0	1	1	0	1
Pennsylvania	1	1	2	0	2
Puerto Rico	1	2	3	0	3
Tennessee	2	1	3	0	3
Texas	1	4	5	0	5
Utah	1	0	1	0	1
Virginia	1	2	3	0	3
Washington	0	1	1	0	1
Wisconsin	3	1	4	0	4

*previously reported in Ferre *et al* (1).
**cohort newly enrolled in the present study.
***race and ethnicity are self-reported.

Supplemental Table 2: Frequency of mutant *AIRE* alleles amongst the American and European APECED patients included in the present study.

Coding DNA Sequence	Protein Sequence	Combined American cohorts* (n=92)<br><br>Number of patients (%)	European cohort** (n=12)<br><br>Number of patients (%)	*AIRE* region
Homozygous for c.967_979del	p.L232Sfs*51	25 (27)	7 (58)	Exon 8
Compound heterozygous for c.967_979del				
and c.769C>T	p.R257*	18 (20)	ND	Exon 6
and c.232T>C	p.T78A	3 (3)	ND	Exon 2
and c.190_226del	p.S64Tfs*71	3 (3)	ND	Exon 2
and c.1163_1164insA	p.M388Ifs*36	2 (2)	ND	Exon 10
and c.1249_1250insC	p.L417Pfs*7	2 (2)	ND	Exon 10
and c.1265delC	p.P422Lfs*58	1 (1)	ND	Exon 10
and c.131A>C	p.Q44P	1 (1)	ND	Exon 1
and c.463G>A	p.G155S	1 (1)	ND	Exon 3
and c.1503+1G>T	n/a	1 (1)	ND	Intron 12
and c.274C>T	p.A92T	1 (1)	ND	Exon 2
and c.1616C>T	p.P539L	1 (1)	ND	Exon 14
and c.132+1_132+3delinsCT	n/a	1 (1)	ND	Intron 1
and c.522_523ins13	p.L323Sfs*51	1 (1)	ND	Exon 8
and c.83T>C	p.L28P	1 (1)	ND	Exon 1
and c.20_115del	p.L7_V38del	1 (1)	ND	Exon 1
and 1781 base pair deletion spanning exon 1 to exon 4	n/a	1 (1)	ND	Exon 1 to 4

Homozygous for c.769C>T	p.R257*	4 (4)	ND	Exon 6
Compound heterozygous for c.769C>T				
and c.789delC	p.A264Lfs*114	2 (2)	ND	Exon 6
and c.1364G>A	p.W455*	1 (1)	ND	Exon 11
Homozygous for c.1504-818 G>A	n/a	8 (9)	ND	Intron 12
Compound heterozygous for c.1504-818 G>A				
and c.958delC	p.L320Wfs*58	1 (1)	ND	Exon 8
and c.1588_*335del	p.Q530Wfs*5	1(1)	ND	Exon 14
Homozygous for c.205_208dupCAGG	p.D70Afs*148	1 (1)	ND	Exon 8
Homozygous for c.328delC	p.R110fs*37	1(1)	ND	Exon 3
Homozygous for c.463+2T>C	p.P166L	ND	3 (25)	Exon 4
Homozygous for c.931delT	p.C311Vfs*67	ND	1 (8)	Exon 8
Homozygous for c.958delC	p.L320Wfs*58	2 (2)	ND	Exon 8
Homozygous for c.1235delC	p.S412fs*68	1 (1)	ND	Exon 10
Compound heterozygous for c.47C>T	p.L323fs*373			Exon 1
and c.1094_1106del	p.T16M	1 (1)	ND	Intron 9 to Exon 10
Compound heterozygous for c.86T>C and large deletion spanning entire *AIRE* gene	p.L29P	1 (1)	ND	Exon 1

Compound heterozygous for c.278T>G and c.834C>G	p.L93R<br>p.S278R	1 (1)	ND	Exon 2<br>Exon 7
Compound heterozygous for c.415C>T and c.1249_1250insC	p.L417Pfs*7<br>pR139*	1 (1)	ND	Exon 10<br>Exon 3
Compound heterozygous for c.242T>C and c.1265delC	p.L81P<br>p.P422fs	ND	1 (8)	Exon 2<br>Exon 10
Heterozygous for[1]<br>c.1322C>T	p.T441M	1 (1)	ND	Exon 11
No identified *AIRE* variants[1]	n/a	1 (1)	ND	n/a

Abbreviations: ND, not detected; n/a, not applicable; [1]These patients met classic diagnostic criteria of APECED (chronic mucocutaneous candidiasis in combination with hypoparathyroidism and/or adrenal insufficiency).
* combination of previously reported patients in Ferre *et al* (1) and newly enrolled patients in the present study.
**cohort newly enrolled in the present study.

Supplemental Table 3: Frequency of endocrine and non-endocrine manifestations in APECED patients from the Americas, Europe, and combined geographic regions.

	Number of patients with indicated number of developed manifestations															
	**0**	**1**	**2**	**3**	**4**	**5**	**6**	**7**	**8**	**9**	**10**	**11**	**12**	**13**	**14**	**15**
**First American cohort* (n=35)**																
**All manifestations**	0	0	1	0	1	3	0	4	6	1	3	4	3	3	5	1
**Endocrinopathies**	0	4	11	11	6	3	0	0	0	0	0	0	0	0	0	0
**Non-Endocrine disease**	0	2	0	4	2	6	2	1	7	3	4	2	2	0	0	0
**Second American cohort** (n=57)**																
**All manifestations**	0	0	2	3	3	10	4	7	4	6	9	5	1	1	1	1
**Endocrinopathies**	4	12	21	11	8	0	1	0	0	0	0	0	0	0	0	0
**Non-Endocrine disease**	0	2	2	10	4	14	7	7	5	4	2	0	0	0	0	0
**European cohort** (n=12)**																
**All manifestations**	**0**	0	0	4	1	1	1	0	4	0	0	0	0	1	0	0
**Endocrinopathies**	**1**	4	3	3	1	0	0	0	0	0	0	0	0	0	0	0
**Non-Endocrine disease**	**0**	0	4	2	2	2	0	1	0	0	1	0	0	0	0	0
**Entire cohort (n=104)**																
**All manifestations**	0	0	3	7	5	14	5	11	14	7	12	9	4	5	6	2
**Endocrinopathies**	5	20	35	25	15	3	1	0	0	0	0	0	0	0	0	0
**Non-Endocrine disease**	0	4	6	16	8	22	9	9	12	7	7	2	2	0	0	0

*previously reported in Ferre *et al* (1).

**cohort newly enrolled in the present study.

Supplemental Table 4: Summary of the presence of autoantibodies (AAB) to IL-17A, IL-17F, and IL-22 in the newly enrolled American and European cohort, their frequency in patients with or without chronic mucocutaneous candidiasis (CMC), and their association with the presence of CMC. n = 68 as AAB data was not available on one patient.

AAB	AAB positivity in patients with CMC % (n)	AAB positivity in patients without CMC % (n)	AAB positivity in all patients % (n)	Association of AAB positivity with presence of CMC (p-value)
IL-17A	32% (19/59)	0% (0/9)	28% (19/68)	0.1081
IL-17F	86% (51/59)	67% (6/9)	84% (57/68)	0.3103
IL-22	88% (52/59)	89% (8/9)	88% (60/68)	0.6241
IL-17A, IL-17F, and/or IL-22	93% (55/59)	89% (8/9)	93% (63/68)	0.8245

Supplemental Table 5: Frequency of non-classic triad clinical manifestations developing before APECED patients reach a classic diagnostic dyad.

	First American cohort* (n=35)	Second American cohort** (n=57)	All Americans combined (n=92)	European cohort** (n=12)	Entire cohort (n=104)
**Development of non-classic triad manifestations before reaching diagnostic dyad**					
Number (percent) of patients who developed non-dyad manifestations before diagnostic dyad	28 (80)	52 (91)	80 (87)	10 (83)	90 (87)
Average (median) number of non-dyad manifestations developed	2.5 (2)	2.1 (1.5)	2.2 (2)	1.9 (1.5)	2.2 (2)
Range of non-dyad manifestations developed	0 to 9	0 to 6	0 to 9	0 to 7	0 to 9
**Number (percent) of specific non-classic triad manifestations developing before diagnostic dyad**					
APECED rash	22 (63)	20 (35)	42 (46)	7 (58)	49 (47)
Autoimmune enteritis	15 (43)	17 (30)	32 (35)	3 (25)	35 (34)
Enamel hypoplasia	12 (34)	16 (28)	28 (30)	5 (42)	33 (32)
Autoimmune hepatitis	4 (11)	6 (11)	10 (11)	1 (8)	11 (11)
Autoimmune pneumonitis	7 (20)	5 (9)	12 (13)	2 (17)	14 (13)
Keratoconjunctivitis	4 (11)	3 (5)	7 (8)	0 (0)	7 (7)
Autoimmune gastritis	1 (3)	2 (4)	3 (3)	0 (0)	3 (3)
B12 deficiency	0 (0)	3 (5)	3 (3)	0 (0)	3 (3)
Sjogren's-like syndrome	7 (20)	0 (0)	7 (8)	0 (0)	7 (7)
Alopecia	2 (6)	8 (14)	10 (11)	2 (17)	12 (12)
Nail dystrophy	4 (11)	4 (7)	8 (9)	2 (17)	10 (10)
Vitiligo	2 (6)	2 (4)	4 (4)	1 (8)	5 (5)
Hypertension	1 (3)	0 (0)	1 (1)	0 (0)	1 (1)
Hypothyroidism	3 (9)	5 (9)	8 (9)	0 (0)	8 (8)
Growth hormone deficiency	1 (3)	0 (0)	1 (1)	0 (0)	1 (1)
Type-1 diabetes	1 (3)	0 (0)	1 (1)	0 (0)	1 (1)

*previously reported in Ferre *et al* (1).
**cohort newly enrolled in the present study.

Supplemental Table 6: Distribution of diseases amongst the first two consecutive manifestations in American and European APECED patients.

Clinical manifestations	1st American cohort* (n=35) Number of patients (%) developing the designated manifestation as:			2nd American cohort** (n=57) Number of patients (%) developing the designated manifestation as:			European cohort** (n=12) Number of patients (%)developing the designated manifestation as:			Entire cohort (n=104) Number of patients (%) developing the designated manifestation as:		
	1st	2nd	1st or 2nd	1st	2nd	1st or 2nd	1st	2nd	1st or 2nd	1st	2nd	1st or 2nd
Chronic mucocutaneous candidiasis	12 (34)	8 (23)	20 (57)	34 (60)	6(11)	40 (70)	7 (58)	1 (8)	8 (67)	53 (51)	15 (14)	68 (65)
APECED rash	13 (37)	2 (6)	15 (43)	12 (21)	7 (12)	19 (33)	4 (33)	2 (17)	6 (50)	29 (28)	11 (11)	40 (38)
Hypoparathyroidism	5 (14)	7 (20)	12 (34)	2 (4)	9 (16)	11 (19)	1 (8)	2 (17)	3 (25)	8 (8)	18 (17)	26 (25)
Autoimmune enteritis	2 (6)	5 (14)	7 (20)	1 (2)	11 (19)	12 (21)	0 (0)	2 (17)	2 (17)	3 (3)	18 (17)	21 (20)
Enamel hypoplasia	1 (3)	3 (9)	4 (12)	3 (5)	8 (14)	11 (19)	0 (0)	3 (25)	3 (25)	4 (4)	14 (13)	18 (17)
Autoimmune pneumonitis	0 (0)	1 (3)	1 (3)	1 (2)	6 (11)	7 (12)	0 (0)	2 (17)	2 (17)	1 (1)	9 (9)	10 (10)
Keratoconjunctivitis	0 (0)	2 (6)	2 (3)	2 (4)	2 (4)	4 (7)	0 (0)	0 (0)	0 (0)	2 (2)	4 (4)	6 (6)
Nail dystrophy	0 (0)	3 (9)	3 (9)	0 (0)	2 (4)	2 (4)	0 (0)	0 (0)	0 (0)	0 (0)	5 (5)	5 (5)
Hypothyroidism	0 (0)	1 (3)	1 (3)	1 (2)	1 (2)	2 (4)	0 (0)	0 (0)	0 (0)	1 (1)	2 (2)	3 (3)
Adrenal insufficiency	0 (0)	1 (3)	1 (3)	1 (2)	1 (2)	2 (4)	0 (0)	0 (0)	0 (0)	1 (1)	2 (2)	3 (3)
Sjogren's-like syndrome	0 (0)	2 (6)	2 (6)	0 (0)	0 (0)	0 (0)	0 (0)	0 (0)	0 (0)	0 (0)	2 (2)	2 (2)
Autoimune hepatitis	0 (0)	0 (0)	0 (0)	0 (0)	1 (2)	1 (2)	0 (0)	0 (0)	0 (0)	0 (0)	1 (1)	1 (1)
Vitiligo	0 (0)	0 (0)	0 (0)	0 (0)	1 (2)	1 (2)	0 (0)	0 (0)	0 (0)	0 (0)	1 (1)	1 (1)
Alopecia	1 (3)	0 (0)	1 (3)	0 (0)	0 (0)	0 (0)	0 (0)	0 (0)	0 (0)	1 (1)	0 (0)	1 (1)
Hypertension	1 (3)	0 (0)	1 (3)	0 (0)	0 (0)	0 (0)	0 (0)	0 (0)	0 (0)	1 (1)	0 (0)	1 (1)
Growth hormone deficiency	0 (0)	0 (0)	0 (0)	0 (0)	2 (4)	2 (4)	0 (0)	0 (0)	0 (0)	0 (0)	2 (2)	2 (2)

*previously reported in Ferre *et al* (1).
**cohort newly enrolled in the present study.

Supplemental Table 7: Absolute number of lymphocyte subsets in the peripheral blood of APECED patients and healthy donors.

Cell Subset	APECED patients (n=100)		Controls (n=40)		
	Absolute number of cell subset per µL of blood				
	Mean	Range	Mean	Range	*p-value*
**$CD3^+$ T cells**	2024	393-4822	1400	529-2536	*<0.0001*
**$CD4^+$ T cells**	1231	44-3387	857.2	273-1808	*<0.0001*
**$CD4^+$ $CD45RA^+$ $CD62L^+$ naïve T cells**	664.8	10-1688	428.5	99-1169	*0.0002*
**$CD4^+$ $CD45RA^-$ $CD62L^+$ central memory T cells**	429.9	26-1112	379.7	122-729	*0.3854*
**$CD4^+$ $CD45RA^-$ $CD62L^-$ effector memory T cells**	119.8	8-800	111.3	38-314	*0.4627*
**$CD4^+$ $CD45RA^+$ $CD62L^-$ effector T cells**	13.8	0-249	7.5	0-90	*0.8996*
**$CD8^+$ T cells**	666.2	53-2429	456.4	143-854	*0.0074*
**$CD8^+$ $CD45RA^+$ $CD62L^+$ naïve T cells**	356.6	13-1135	241.8	67-600	*0.0043*
**$CD8^+$ $CD45RA^-$ $CD62L^+$ central memory T cells**	77.4	1-400	79	15-202	*0.2256*
**$CD8^+$ $CD45RA^-$ $CD62L^-$ effector memory T cells**	110.2	4-773	74.8	15-192	*0.5266*
**$CD8^+$ $CD45RA^+$ $CD62L^-$ effector T cells**	121.7	5-1568	71.3	11-282	*0.7365*
**CD4/CD8 ratio**	2.533	0.02000-14.43	2.155	0.61-6.1	*0.2958*
**$CD3^+$ $CD4^-$ $CD8^-$ double-negative T cells**	125.8	7-1435	70.5	18-233	*0.0003*
**$CD20^+$ B cells**	298.7	0-1507	179.6	39-480	*0.3405*
**$CD20^+$ $CD27^+$ memory B cells**	48	0-264	36	12-109	*>0.9999*
**$CD20^+$ $CD38^+$ transitional B cells**	255.5	0-1423	165.2	31-456	*0.7365*
**$CD20^+$ $CD10^+$ immature B cells**	74.2	0-615	48.7	8-170	*0.5014*
**$CD21^+$ $CD10^+$ immature B cells***	48.5	0-447	30.7	1-111	*0.3069*
**$CD20^+$ IgM^- $CD38^{hi}$ plasmablasts**	1.1	0-7	0.2	0-3	*0.0012*
**$CD20^+$ IgM^+ $CD10^+$ immature B cells**	52.8	0-429	42.3	6-142	*0.2058*

**CD20^{+} CD38^{+} CD10^{+} early transitional naïve B cells**	55.1	0-452	45.9	5-163	*0.1775*
**CD20^{+} CD27^{+} IgM^{+} memory non-switched B cells**	20.9	0-137	14.7	2-49	*0.6042*
**CD20^{+} CD27^{+} IgM^{-} memory switched B cells**	18.4	0-77	15.3	1-52	*0.4348*
**CD19^{+} CD21^{lo} CD38^{lo} autoreactive B cells***	31.8	0-361	7.5	1-46	*<0.0001*
**CD3^{-} CD56^{+} NK cells**	175.7	4-1078	335.9	121-863	*<0.0001*
**CD3^{+} CD56^{+} NKT cells**	208.6	31-2087	127.8	28-451	0.0262

The mean and range of lymphocyte subsets are presented for peripheral blood drawn from patients with APECED (n=100; age range 2-66 years; 59% female) or unaffected controls (n=40; age range 23-62 years; 54% female). *P* values were calculated using the Mann-Whitney Test. *Due to available data, analysis of lymphocyte subsets of patients with APECED included 98 patients when evaluating CD21^{+}CD10^{+} immature B cells and included 97 patients when evaluating autoreactive CD19^{+}CD21^{lo}CD38^{lo} B cells.

Supplemental Table 8: Percent of lymphocyte subsets within corresponding lymphocytes in the peripheral blood of APECED patients and healthy donors.

Cell Subset	APECED patients (n=100)		Controls (n=40)		
	% of cell subset within corresponding lymphocytes				
	Mean	Range	Mean	Range	*p-value*
**$CD4^+$ T cells (within $CD3^+$ cells)**	62.1	1.7-98.7	61.2	32.9-81.3	*0.4021*
**$CD8^+$ T cells (within $CD3^+$ cells)**	31.6	6.3-95.4	37.6	15.8-66.4	*0.0005*
**Naïve T cells (within $CD4^+$ cells)**	52.25	5.0-85.0	43.9	20.7-70.2	*0.0045*
**Central memory T cells (within $CD4^+$ cells)**	36.2	7.3-67.3	41.9	22.1-62.7	*0.016*
**Effector memory T cells (within $CD4^+$ cells)**	10.3	2.3-52.9	13.2	3.9-31.1	*0.0005*
**Effector T cells (within $CD4^+$ cells)**	1.1	0-12.6	1.1	0-7.2	*0.0354*
**Naïve T cells (within $CD8^+$ cells)**	54.9	8.1-90.6	38.3	0-64	*<0.0001*
**Central memory T cells (within $CD8^+$ cells)**	12.6	0.1-43.1	12	0-24.2	*0.9606*
**Effector memory T cells (within $CD8^+$ cells)**	17.2	0.2-61.9	39.1	18.1-100	*<0.0001*
**Effector T cells (within $CD8^+$ cells)**	15.3	1.8-64.6	10.6	0-30.8	*0.1674*
**Double-negative T cells (within $CD3^+$ cells)**	6.1	0.6-39.9	3.8	1.1-10.4	*<0.0001*
**$CD20^+$ $CD27^+$ memory B cells (within CD20+ cells)**	23.5	0-100	22.9	7.1-65.1	*0.4007*
**$CD20^+$ $CD38^+$ transitional B cells (within CD20+ cells)**	78.8	0-100	91.3	79.5-100	*0.0018*
**$CD20^+$ $CD10^+$ immature B cells (within CD20+ cells)**	21.8	0-100	26.6	14.9-44.8	*0.004*
**$CD21^+$ $CD10^+$ immature B cells (within CD20+ cells)***	11.5	0-63.2	15.9	2.2-36.9	*0.0005*
**$CD20^+$ IgM^- $CD38^{hi}$ plasmablasts (within CD20+ cells)**	0.3	0-3.6	0.1	0-0.9	*0.0025*
**$CD20^+$ IgM^+ $CD10^+$ immature B cells (within CD20+ cells)**	14.6	0-63.2	22.7	12.8-39.1	*<0.0001*
**$CD20^+$ $CD38^+$ $CD10^+$ early transitional naïve B cells (within CD20+ cells)**	15.4	0-63.2	24.8	10.9-44.3	*<0.0001*

**CD20^{+} CD27^{+} IgM^{+} memory non-switched B cells (within CD20+ cells)**	10	0-72.7	10.3	1.9-43.5	*0.5789*
**CD20^{+} CD27^{+} IgM^{-} memory switched B cells (within CD20+ cells)**	7.5	0-45.9	9.1	1.4-21.1	*0.0163*
**CD19^{+} CD21^{lo} CD38^{lo} autoreactive B cells (within CD19+ cells)***	16.3	0-76.9	4.3	1-17.7	*<0.0001*

The mean and range of the percent of lymphocyte subsets within the indicated lymphocyte set are presented for peripheral blood drawn from patients with APECED (n=100; age range 2-66; 59% female) or unaffected controls (n=40; age range 12-62 years; 54% female). *P* values were calculated using the Mann-Whitney Test. *Due to available data, analysis of lymphocyte subsets of patients with APECED included 98 patients when evaluating CD21^{+}CD10^{+} immature B cells and included 97 patients when evaluating autoreactive CD19^{+}CD21^{lo}CD38^{lo} B cells.

Supplemental Figure 1. Time to development of APECED manifestations by the presence of the c.967_979del variant in the new cohort (n=69). Kaplan-Meier curves are shown illustrating the relationship between carrying c.967_979del in homozygosity (group A), carrying a heterozygous c.967_979del variant in *AIRE* (Group B), and carrying no c.967_979del variant in *AIRE* (Group C) with the time to development of A) autoimmune pneumonitis, B) autoimmune hepatitis, C) APECED rash, D) Sjogren's-like syndrome, or E) autoimmune gastritis. P_1 represents the *P* value comparing groups A and B; P_2 represents the *P* value comparing groups A and C; P_3 represents the *P* value comparing groups B and C. *P* values are based on the log-rank test.

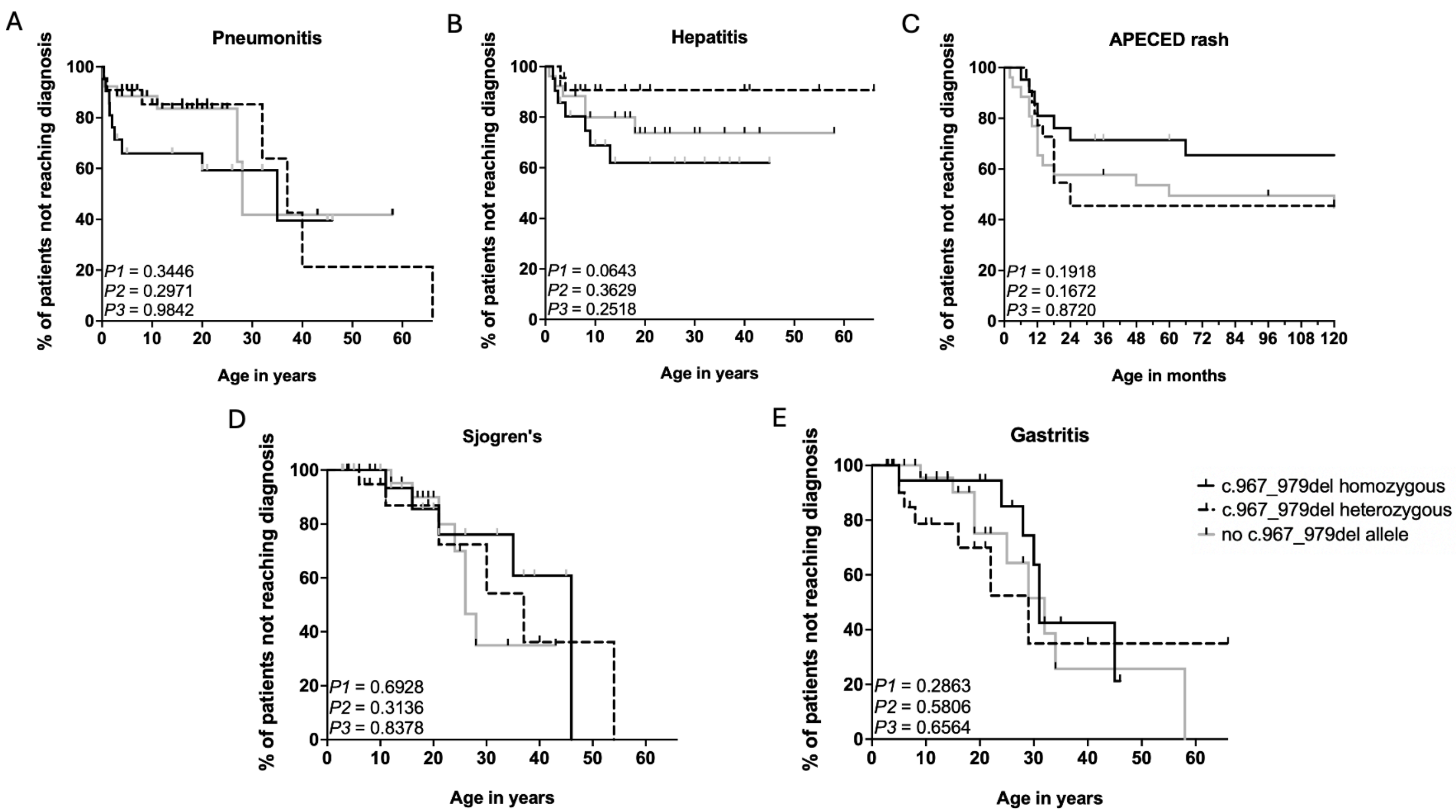

Supplemental Figure 2. Time to development of APECED manifestations by the presence of autoantibodies.
Kaplan-Meier curves are shown illustrating the relationship between the presence of targeted autoantibodies and the time to development of APECED manifestations for the 69 newly enrolled APECED patients reported in this study and the entire cohort of 104 APECED patients. Autoantibody and time to development relationships shown here are for A) anti-IL17A and chronic mucocutaneous candidiasis (CMC), B) anti-17F and CMC, C) anti-IL22 and CMC, D) anti-21 hydroxylase and adrenal insufficiency, E) anti-intrinsic factor and B12 deficiency, F) anti-thyroglobulin and hypothyroidism, G) anti-thyroid peroxidase and hypothyroidism, H) anti-BPIFP1 and autoimmune pneumonitis, and I) anti-KCNRG and autoimmune pneumonitis. *P* values are based on the log-rank test. n indicates the number of patients included in each graph. Though clinical evaluation occurred in all patients, the number of patients in each graph varies dependent upon the number of patients who had the corresponding autoantibody testing performed.

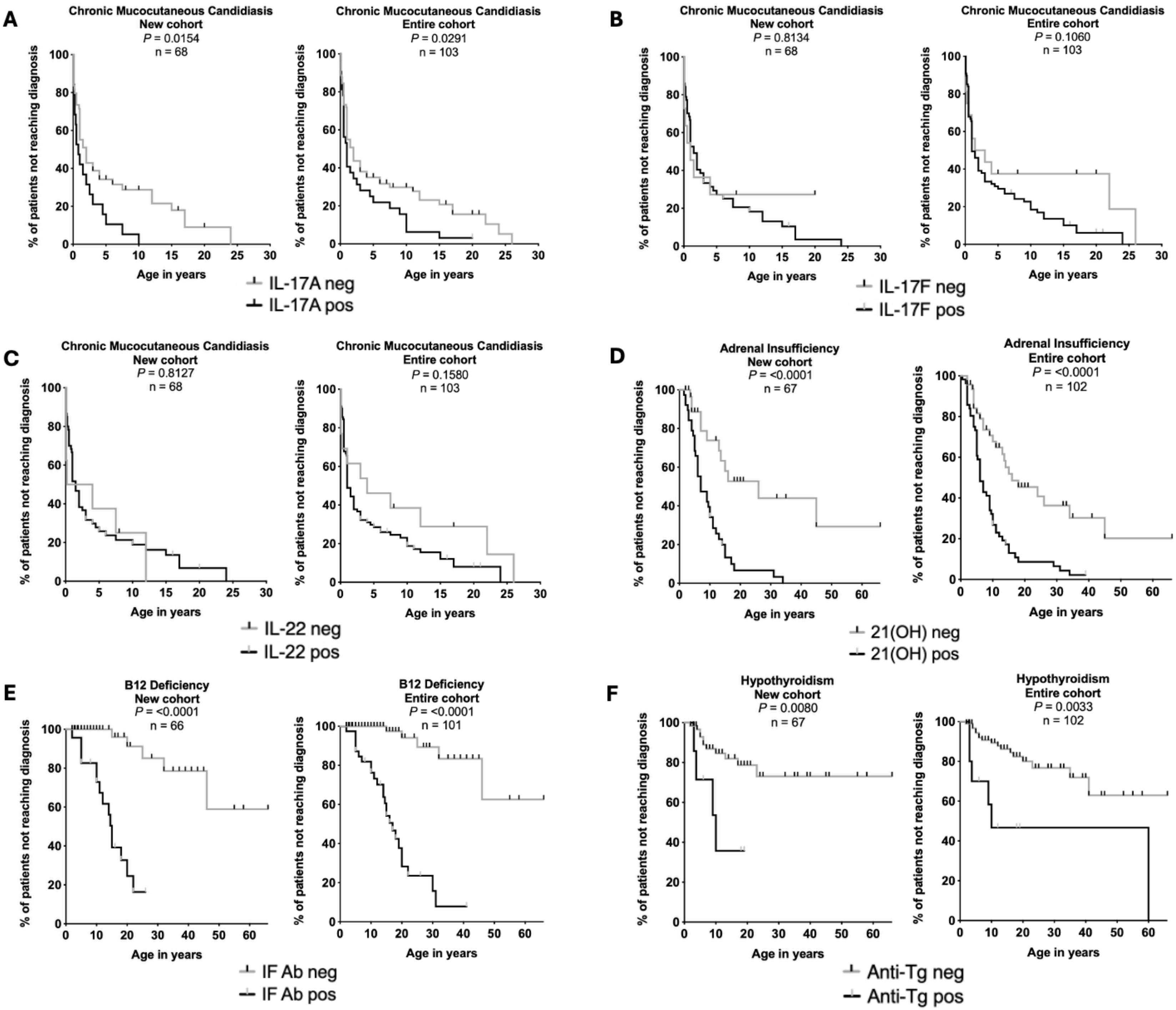
A
Chronic Mucocutaneous Candidiasis
New cohort
P = 0.0154
n = 68
Chronic Mucocutaneous Candidiasis
Entire cohort
P = 0.0291
n = 103
IL-17A neg
IL-17A pos
B
Chronic Mucocutaneous Candidiasis
New cohort
P = 0.8134
n = 68
Chronic Mucocutaneous Candidiasis
Entire cohort
P = 0.1060
n = 103
IL-17F neg
IL-17F pos
C
Chronic Mucocutaneous Candidiasis
New cohort
P = 0.8127
n = 68
Chronic Mucocutaneous Candidiasis
Entire cohort
P = 0.1580
n = 103
IL-22 neg
IL-22 pos
D
Adrenal Insufficiency
New cohort
P = <0.0001
n = 67
Adrenal Insufficiency
Entire cohort
P = <0.0001
n = 102
21(OH) neg
21(OH) pos
E
B12 Deficiency
New cohort
P = <0.0001
n = 66
B12 Deficiency
Entire cohort
P = <0.0001
n = 101
IF Ab neg
IF Ab pos
F
Hypothyroidism
New cohort
P = 0.0080
n = 67
Hypothyroidism
Entire cohort
P = 0.0033
n = 102
Anti-Tg neg
Anti-Tg pos
% of patients not reaching diagnosis
Age in years

G

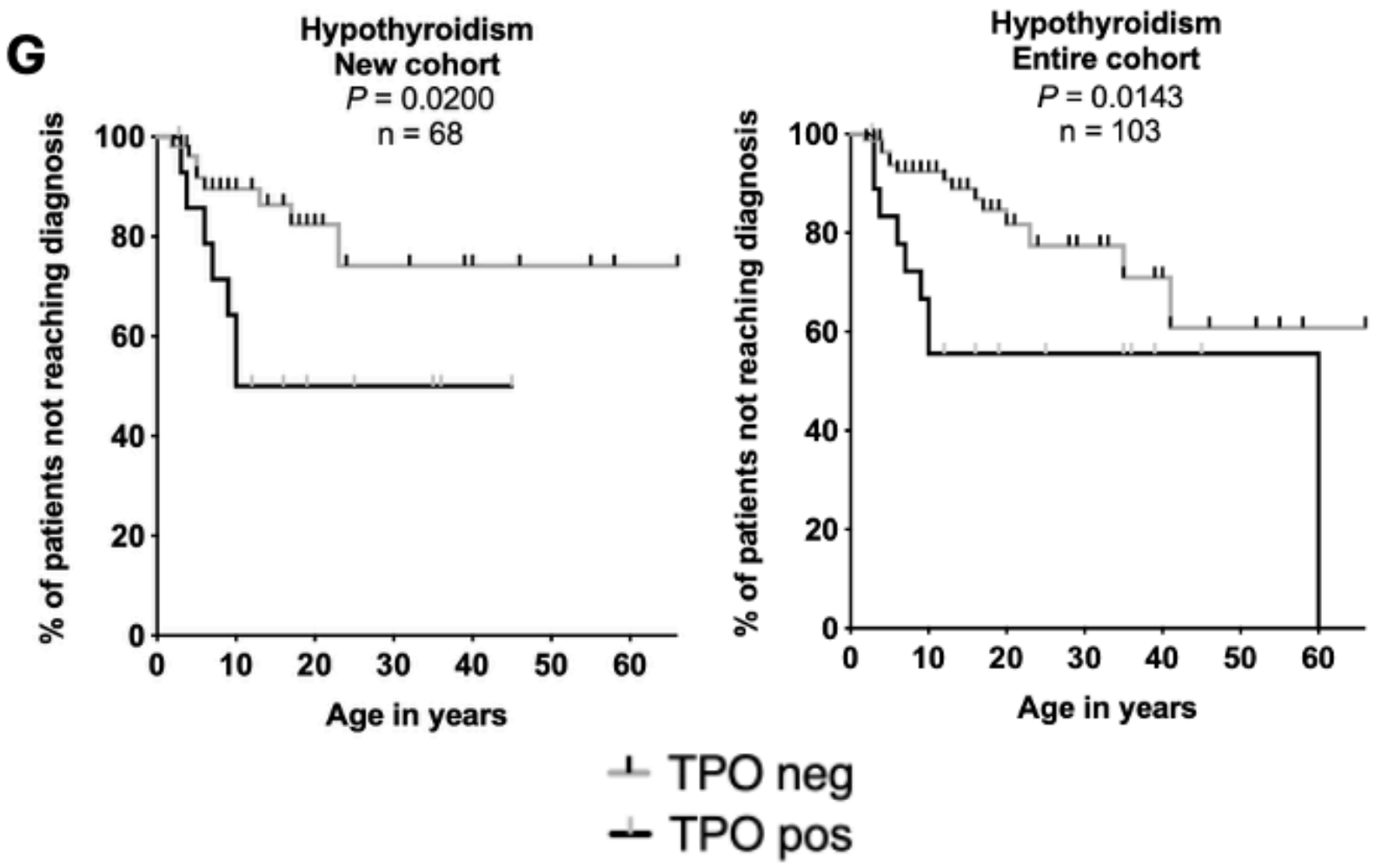
Hypothyroidism
New cohort
P = 0.0200
n = 68
Hypothyroidism
Entire cohort
P = 0.0143
n = 103
% of patients not reaching diagnosis
Age in years
TPO neg
TPO pos

H

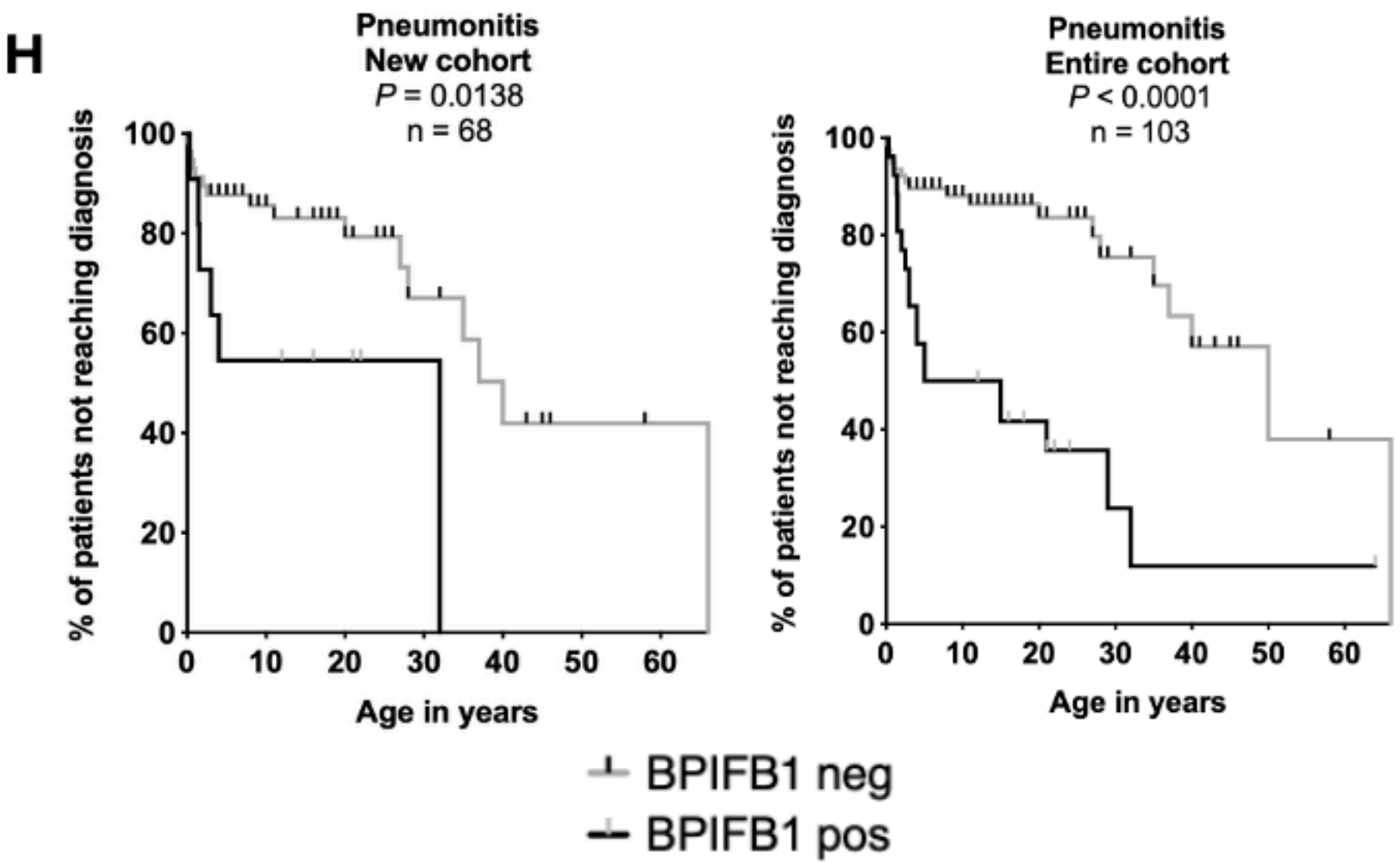
Pneumonitis
New cohort
P = 0.0138
n = 68
Pneumonitis
Entire cohort
P < 0.0001
n = 103
% of patients not reaching diagnosis
Age in years
BPIFB1 neg
BPIFB1 pos

I

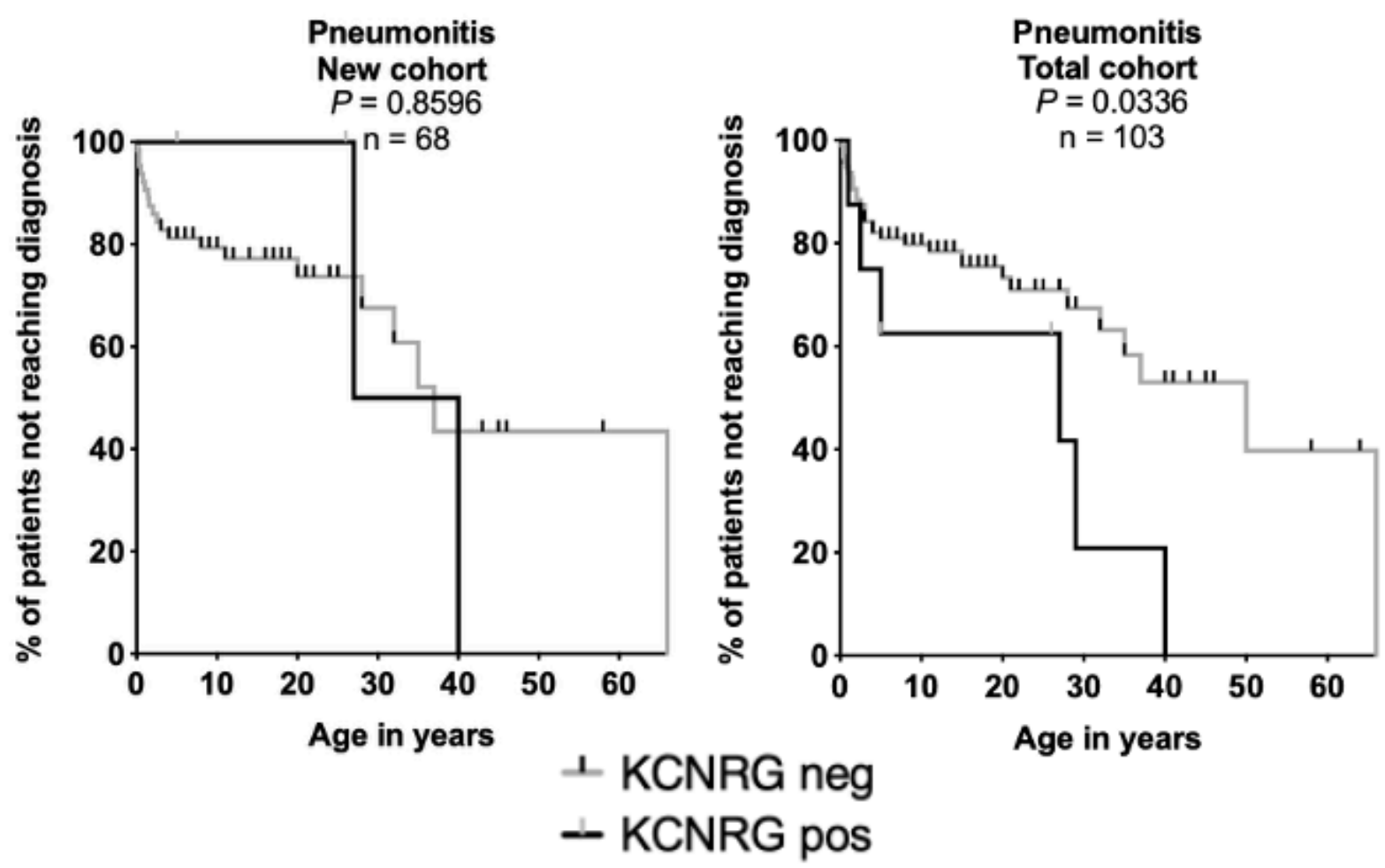
Pneumonitis
New cohort
P = 0.8596
n = 68
Pneumonitis
Total cohort
P = 0.0336
n = 103
% of patients not reaching diagnosis
Age in years
KCNRG neg
KCNRG pos

Supplemental Figure 3. Serum CXCL9 levels are elevated across patient cohorts and compared with healthy control. CXCL9 was measured via ELISA from available sera of healthy donors (n = 47), the initial cohort (n = 31), and the newly enrolled cohort (n = 65). ****P* = 0.0004; *****P* < 0.0001 via the Kruskal-Wallis test with Dunn's multiple comparasons. Serum CXCL9 measurements from a subset of healthy donors and patients were previously reported (2); these data are included here alongside newly analyzed samples and presented stratified by patient cohort.

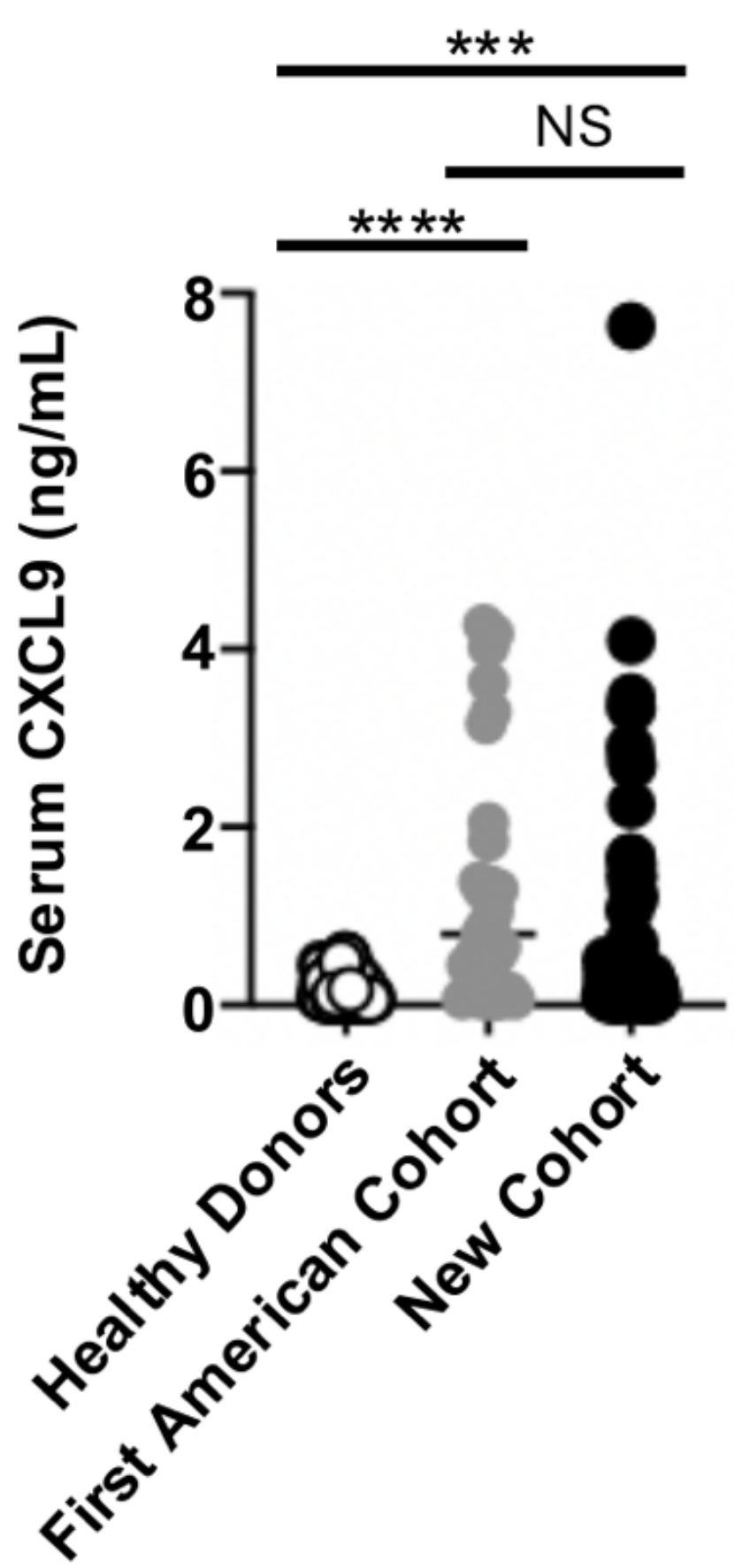

References

1. Ferre EM, Rose SR, Rosenzweig SD, Burbelo PD, Romito KR, Niemela JE, et al. Redefined clinical features and diagnostic criteria in autoimmune polyendocrinopathy-candidiasis-ectodermal dystrophy. *JCI Insight.* 2016;1(13).
2. Oikonomou V, Smith G, Constantine GM, Schmitt MM, Ferre EMN, Alejo JC, et al. The Role of Interferon-gamma in Autoimmune Polyendocrine Syndrome Type 1. *N Engl J Med.* 2024;390(20):1873–84.
